# Coordination through databases can improve prescribed burning as a conservation tool to promote forest biodiversity

**DOI:** 10.1007/s13280-017-0987-6

**Published:** 2017-11-10

**Authors:** Ellinor Ramberg, Joachim Strengbom, Gustaf Granath

**Affiliations:** 1 Department of Ecology, Swedish University of Agricultural Sciences, Box 7044, 750 07 Uppsala, Sweden; 2 Present Address: Department of Plant Ecology and Evolution, Evolutionary Biology Centre, Uppsala University, Norbyvägen 18D, 752 36 Uppsala, Sweden

**Keywords:** Boreal, Fire-dependent species, Forest management, *Geranium lanuginosum*, Prescribed burning, *Stephanopachys substriatus*

## Abstract

Prescribed fires are a common nature conservation practice. They are executed by several parties with limited coordination among them, and little consideration for wildfire occurrences and habitat requirements of fire-dependent species. Here, we gathered data on prescribed fires and wildfires in Sweden during 2011–2015 to (i) evaluate the importance and spatial extent of prescribed fires compared to wildfires and (ii) illustrate how a database can be used as a management tool for prescribed fires. We found that on average only 0.006% (prescribed 65%, wildfires 35%) of the Swedish forest burns per year, with 58% of the prescribed fires occurring on clearcuts. Also, both wildfires and prescribed fires seem to be important for the survival of fire-dependent species. A national fire database would simplify coordination and make planning and evaluation of prescribed fires more efficient. We propose an adaptive management strategy to improve the outcome of prescribed fires.

## Introduction

Wildfires have historically been a major disturbance in the fennoscandian boreal forests, with profound influence on the structuring and functioning of these ecosystems (Zackrisson 1977; Niklasson and Granström 2000; Drobyshev et al. 2014). However, increased human population and an expanding forestry industry over the past 150 years have resulted in efficient fire suppression, and fire is no longer a central component of the natural forest dynamics (Niklasson and Granström 2000; Granström and Niklasson 2008). This change in dynamics is considered a major threat to forest biodiversity; however, during the last few decades a growing awareness of the importance of fire for maintaining biodiversity has led to implementation of prescribed fires as a conservation measure (Granström 2001; Niklasson and Drakenberg 2001; Nilsson 2005).

Globally, prescribed forest fires are commonly used to mitigate the effects of wildfires by preventing fuel accumulation. However, there are many examples where prescribed burning is used to maintain fire-dependent ecosystems and biodiversity (e.g., longleaf pine savanna in southeast US, Ryan et al. 2013; Mediterranean shrubland, Fernandez et al. 2013), and in Northern Europe, prescribed burning is used solely for such nature conservation purposes (Finney et al. 2005; Nilsson 2005). In Sweden, prescribed burns are primarily executed by forestry companies and county administrative boards. For forest companies, implementation of prescribed burning is driven by demand from certification schemes such as the Forest Stewardship Council (FSC). Fires are administered both on clearcuts and on standing forest. To comply with FSC certification regulations, companies are required to burn an area corresponding to at least 5% of the harvested forest area over a 5-year period (FSC 2010). In addition to certification-driven incentives, prescribed burning is used to meet the national environmental objectives concerning forest biodiversity, and prescribed fires are executed within protected forest areas by the County Administrative Boards (Nilsson 2005). However, forest companies and the administrative county board do not operate jointly when performing prescribed burnings. Moreover, both parties utilize individual registration and evaluation systems, making the assessment of prescribed fire effects on wildlife not straightforward. A joint database has long been discussed to aid in planning and evaluation of fires, but has to date not been implemented.

The conservation value of prescribed burning has been questioned, as both location choice and execution method of fires may, from a conservation point of view, be suboptimal (Granström 2001; Wikars 2004). Forest companies burn both on clearcuts and on standing forest, and the value of burning clearcuts may be limited due to the small amounts of dead wood it creates. Given that the conservation value of prescribed burning tends to increase with the pre-fire standing tree volume (Hyvärinen et al. 2009; Heikkala et al. 2016), such fires may be less effective in favoring species that are directly dependent on burned wood (Ranius et al. 2014) and the conservation value of prescribed burning tends to increase with the pre-fire standing tree volume (Hyvärinen et al. 2009; Heikkala et al. 2016). Moreover, the vegetation succession following burns on clearcuts differs from that of unlogged sites, or those where some trees are retained for conservation purposes (Johnson et al. 2014). In addition to pre-fire tree volume, it is also highlighted that fires need to be executed based on the requirements (e.g., burn severity) and presence (i.e., spatial proximity) of fire-dependent species if they are to have any discernible effect as a conservation measure (Risberg and Granström 2012; Ranius et al. 2014). Burn severity and location of the prescribed fire in the landscape are largely determined by safety precautions that minimize the risk of losing control and endangering nearby settlements. As a result, prescribed burns are often executed at “suitable” sites and under high moisture conditions which consequently result in marginal effects on the forest landscape (Granström 2001; Wikars 2004). Therefore, to implement long-term management strategies at the landscape level, we require essential knowledge on the type of vegetation that burns and its spatial distribution in the landscape.

There are at least a hundred fire-dependent species in Sweden today, of which many are threatened (Gärdenfors 2015). These species possess highly specialized fire adaptations which reflect a historic fire regime where fire once played a more prominent role in the forest landscape (Wikars 1997; Granström 2001; Risberg 2015). Generally, fire-dependent species have specific requirements regarding the character of the fire which can be divided into two categories: mobile species, which require recently burned areas within their dispersal range, and more or less stationary species which depend on recurring fires within the same area during a specific time period (Granström 2001). Two typical examples for the two categories, respectively, are the mobile beetle *Stephanopachys substriatus* (Paykull) and the stationary herb *Geranium lanuginosum* Lam. In this paper, we use these two red-listed and fire-dependent species to illustrate a proof of concept of the value and use of species-specific information in the fire management decision process.

*Stephanopachys substriatus* is listed as Near Threatened in the Swedish Red List, and also listed in the EU’s habitat directive, making conservation of the species a priority (Gärdenfors 2015). This beetle prefers fire-scarred trees, usually inhabiting them as long as the tree is alive, with beetle larvae developing in the cambium region. They require recently burned wood every 1–10 years and have a dispersal range of around 10 km (Wikars 2004; Ranius et al. 2014). Therefore, to benefit *S. substriatus*, fires need to occur every 1–10 years within a 10-km radius.

*Geranium lanuginosum* is categorized as Endangered on the latest Swedish red list due to a fragmented and severely reduced population (Gärdenfors 2015). *G. lanuginosum* grows mainly on soils that are nutrient rich, with seeds that have short dispersal capabilities and lie dormant in seed banks with germination activated only by temperatures between 50 and 100 °C (Granström and Schimmel 1993). The species is annual but it has been observed that the seeds can lie dormant for at least 200 years and still be viable (Risberg and Granström 2012). *G. lanuginosum* requires recurring fires on the same site within at least 200 years to reproduce.

The objectives of the study were to (i) evaluate the importance for burnt substrate and the spatial extent of prescribed fires compared to wildfires by gathering data on wildfires and prescribed fires in Sweden between 2011 and 2015, and (ii) illustrate how a database can be used as a tool for planning and evaluating prescribed fires at the landscape level using the two species *S. substriatus* and *G. lanuginosum*, as a proof of concept.

## Materials and methods

### Forest fires in Sweden 2011–2015

#### Wildfires

Occurrences and the extent of wildfires were obtained from the Swedish Civil Contingencies Agency, Sweden’s emergency services which registers all wildfires including those affecting forest land. The data extracted included information on the area and coordinates of wildfires over 5 years (2011–2015), as well as if the fires had affected productive forest land or low productive forest land. Fires with an area smaller than 0.5 ha were excluded as their effect on forest land was deemed minimal, and therefore also their significance for fire-dependent species. A similar threshold for fire perimeters has been used in earlier studies comparing wildfires and prescribed fires (Wikars 2004).

#### Prescribed fires

The four largest forestry companies in Sweden that are FSC certified, namely SCA AB, Holmen Skog AB, Sveaskog AB, and Bergvik AB, were approached for data on prescribed fires. Besides these companies, a few landowners occasionally perform prescribed burns but their contribution is considered negligible. The forest companies approached provided data on coordinates and the area of prescribed fires between 2011 and 2015, classified into fires executed on standing forest and those executed on clearcuts. Additional data on prescribed fires (coordinates and area burnt) were collected from the county administrative boards; of Sweden’s 21 counties, 19 responded to our request. The two counties that did not respond had, nonetheless, data on prescribed fires published on their websites, which were downloaded. In total, 15 counties had performed prescribed fires between 2011 and 2015.

### Forest definitions

There were some discrepancies in the definition of forest land between the different involved parties. It was, therefore, necessary to decide on a definition of forest land which was suitable for the data used in the study. In this study, forest land therefore comprises both productive forest land (tree growth ≥ 1 m^3^ wood per hectare and year, tree height ≥ 5 m, crown cover ≥ 10%) and low productive forest land (tree growth < 1 m^3^ per hectare and year, tree height between 0.5 and 5 m, crown cover between 5 and 10%), which follows the Swedish Forest Agency’s definition (Swedish Forest Care Act § 2). Of the 30 536 000 ha of forest land in Sweden, productive forest encompasses 23 429 000 ha, and low productive forest 7 107 000 ha (Fridman et al. 2014; Skogsdata 2016).

### Species distribution data

Species distribution data for *G. lanuginosum* and *S. substriatus* were extracted from the Swedish LifeWatch analysis portal (https://www.analysisportal.se/). The search criteria included all species observation databases connected to the portal. These data originate from different sources (e.g., agency employees, researchers, and citizens) and vary between species in terms of spatial and temporal cover. Although this database may provide limited data for some species at the moment, data quality and the extent of species cover are expected to improve with time.

### Analyses

All coordinates were transformed into SWEREF 99 TM projection for visualization and analysis in ESRI’s ArcGIS application ArcMap. The distribution of each species was mapped together with data for wildfires and prescribed fires. To analyze the spatial relationship between mapped fires and species distribution, the ArcMap analysis tool Proximity was utilized. For *G. lanuginosum*, the Proximity tool Generate Near Table was used to evaluate the spatial relationship between mapped fires and species observations reported between 2011 and 2015. For both *G. lanuginosum* and *S. substriatus*, the Proximity tool Near was used to analyze the distance between mapped prescribed fires between 2011 and 2015 and reported species finds between the periods of 1900–2015 for *G. lanuginosum* and 2005–2015 for *S. substriatus*. We used a longer time span for *G. lanuginosum* to account for its potentially long-lasting seed bank, and shorter time span for *S. substriatus* for which we assumed they can survive in an area for at least 10 years after it was recorded. When analyzing the distance to a fire, we mapped fires as point features instead of polygon features. Using the center and not the edge of the burnt area, the distance from a species record to the nearest fire may be somewhat overestimated. To compensate for this in the analyses, 0.5 km was added to the radius perimeters as the fires were in general small. Based on the species dispersal capabilities (see the Introduction section), the radius search perimeter for *G. lanuginosum* was set to 0.5 km and to 10.5 km for *S. substriatus*.

## Results

### Importance of prescribed fires

The total forest area burned during the study period (2011–2015) was 20 442 ha, an average of 4088 ha annually: 0.013% of Sweden’s forest land (Table 1). A large wildfire in Southern Sweden (in Västmanland county, hereafter called “Vstml fire”) in the summer of 2014, in which 11 070 ha of forest burnt, had a significant impact on the results. Without this fire, the total forest area burnt amounted to 9372 ha, an annual average of 1874 ha: 0.006% of Sweden’s total forest area. With the exception of year 2014, prescribed fires account for 65% of the total burnt forest area annually on average, with the remaining 35% attributed to wildfires. Forestry companies were responsible for 86% of the prescribed fires, of which 67% (i.e., 58% of total area burned annually by prescribed fires) were conducted on clearcut forest sites (Table 1).

**Table 1:** Forest area burnt in hectares and number of fires (in brackets) in Sweden per year (2011–2015), and separated into wildfires and prescribed fires. Wildfires and prescribed fires are further classified based on the forest type: productive and low productive forest. The prescribed fires category also distinguishes between the different stakeholders that execute fires: forestry companies and county boards. The last column indicates the total area burnt without an unusually large wildfire that occurred in year 2014

Fire category, the type of forest impacted and involved stakeholders	2011	2012	2013	2014	2015	Total	Total without Vstml fire 2014
Wildfires							
Productive forest	315 (84)	91 (29)	441 (115)	10 435 (189)	239 (72)	11 521 (489)	1945 (488)
Low productive forest	285 (74)	69 (32)	270 (135)	2080 (148)	76 (42)	2780 (431)	1286 (430)
Total	600 (156)	160 (61)	711 (245)	12 515 (330)	315 (108)	14 301 (900)	3231 (899)
Prescribed fires							
Forestry companies							
Productive forest-clear cuts	1152 (60)	485 (28)	317 (22)	1213 (68)	388 (30)	3555 (208)	3555 (208)
Productive forest-standing forest	259 (31)	83 (17)	444 (51)	742 (69)	197 (14)	1725 (182)	1725 (182)
Low productive forest	0	0	0	0	0	0	0
County boards	76 (7)	119 (11)	157 (19)	117 (13)	392 (26)	861 (76)	861 (76)
Total	1487 (98)	685 (56)	918 (92)	2072 (150)	977 (70)	6 141 (466)	6 141 (466)

All prescribed fires performed by the forest companies were on productive forest land, while no distinction between forest types was made in the county-administered fires. Wildfires burnt a larger area of productive versus low productive forest land. Without the large Vstml fire, 60% of the area burnt was on high productive forest land (81% with the Vstml fire) (Table 1).

**Fig. 1:**
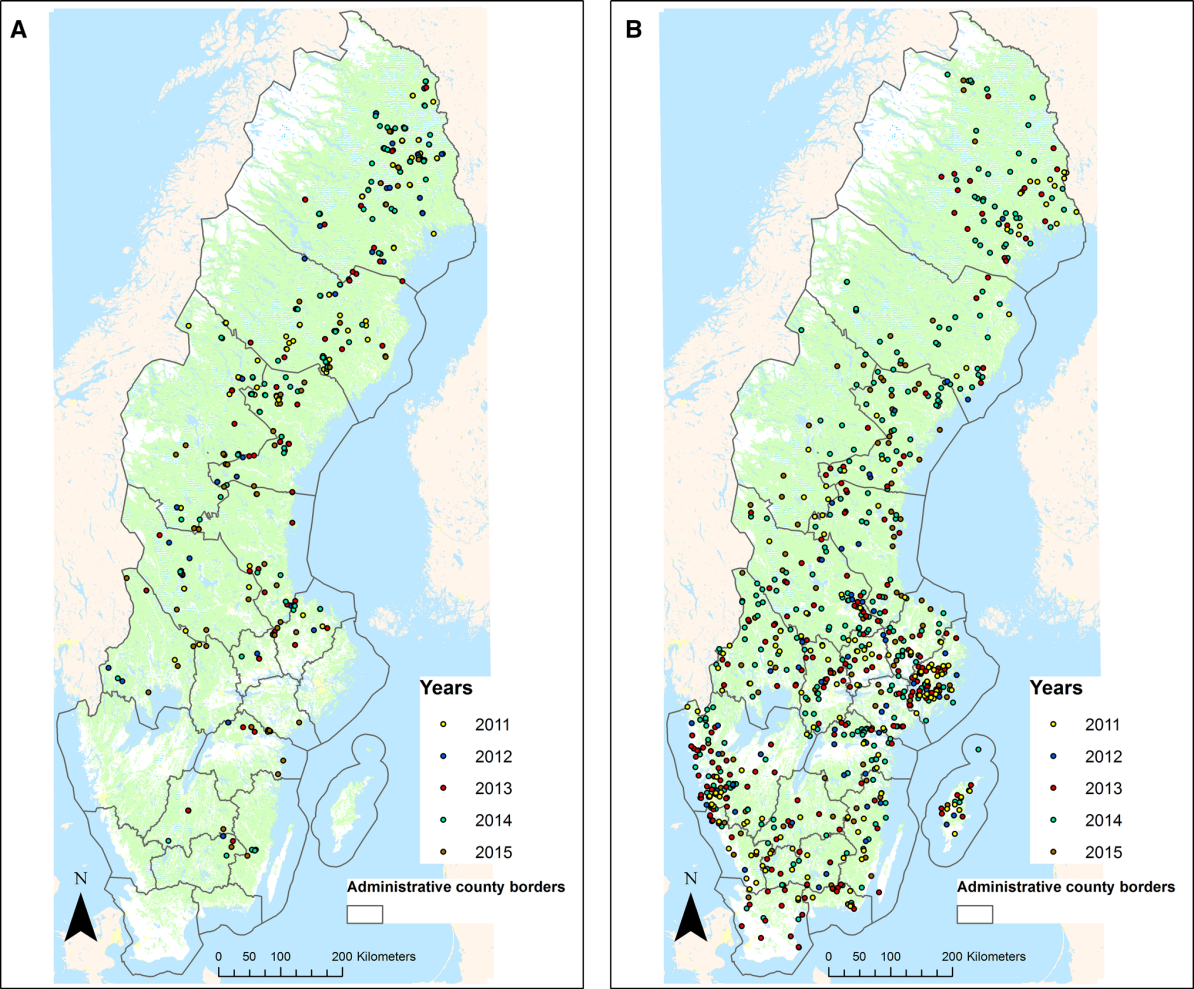
**a** Prescribed fires executed by forest companies and county boards during 2011–2015 in Sweden. **b** Wildfires (≥ 0.5 ha) registered by the Swedish Civil Contingencies Agency during 2011–2015

In general, fires occurring during the study period were small, with fires larger than 20 ha being rare. Prescribed fires had a mean size of 12 ha (median = 7.5 ha). Wildfires seldom affected a larger forest area, with a mean area of 3.5 ha (median = 1 ha). However, wildfires were more common than prescribed fires (Table 1; Fig. 1), and this difference would be even greater if wildfires <0.5 ha were included in the analyses. For both wildfires and prescribed fires, the number of fires and area burnt peaked in 2014 and was the lowest in 2012. Wildfires were relatively evenly spread throughout the country, with a clear clustering of fires in the vicinity of the major cities (e.g., Stockholm and Gothenburg, Fig. 1b). Prescribed fires were more sparsely spread out and predominantly located in the northern part of the country (Fig. 1a).

### Fire-dependent species distribution and forest fires

**Fig. 2:**
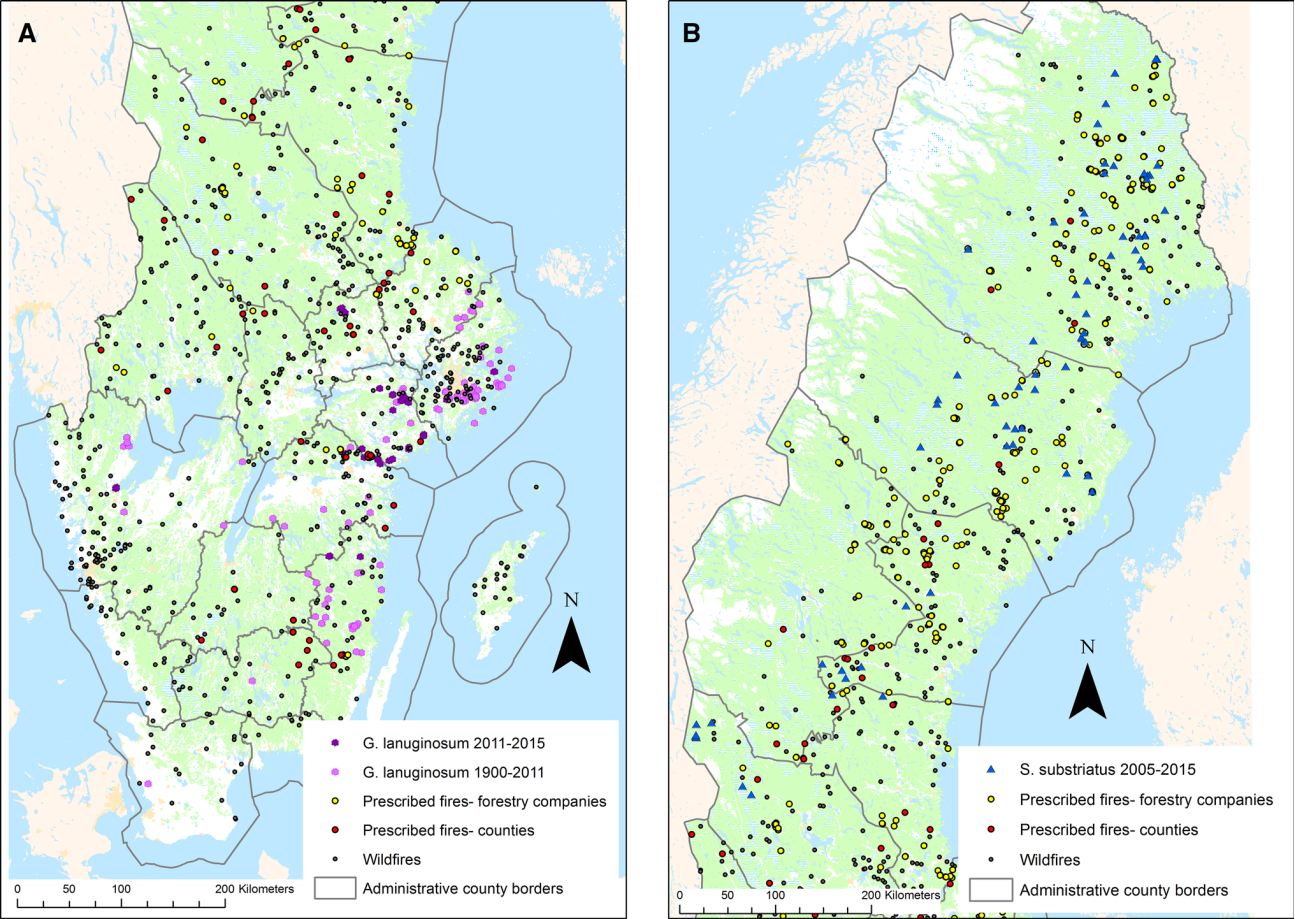
**a** Reported finds of *Geranium lanuginosum* between 1900 and 2015 in relation to wildfires and prescribed fires during 2011–2015 in Sweden. **b** Reported finds of *Stephanopachys substriatus* between 2005 and 2015 in relation to wildfires and prescribed fires during 2011–2015 in Sweden

The distribution of *G. lanuginosum* is confined to southeastern Sweden, which also was demonstrated in the reported finds (Fig. 2a). Between 2011 and 2015, 104 finds of the species were reported. Of the 104 reported finds, only 23 were closer to a prescribed burn than a wildfire. Between 1900 and 2015, 282 finds of *G. lanuginosum* were reported. Of the 282 reported finds, only 17 were within 0.5 km of a prescribed fire executed during 2011–2015. All 17 were within one nature reserve, Fjallmossen, where the county boards had carried out planned fires in 2011, 2013, and 2015.

The beetle *S. substriatus’* distribution area encompasses the northern part of Sweden (Fig. 2b). In total, 81 finds of the species were reported between 2005 and 2015. Of the 81 finds, 37 were within 10.5 km of a mapped prescribed fire, whereof 31 were closest to a prescribed fire executed by forest companies during 2011–2015. The remaining six finds were closest to a prescribed fire executed by county boards.

## Discussion

### Importance of prescribed fires

The average annual forest area burned during the study period was 0.013%, but this result is mainly driven by one single fire, the Vstml fire in 2014. This wildfire was unusually large, 100 times larger than any other fire during the study period, and therefore the long-term annual average proportion of forest land that burns in Sweden is likely closer to 0.006 than 0.013%. The area of forest land that burns today is thereby only a fraction of what has probably burnt historically (Zackrisson 1977; Niklasson and Granström 2000), and in Sweden today around 65% of the burnt forest area per year is a result of prescribed burning. With the majority of today’s burnt substrate in the landscape being created by prescribed burning, it is important that optimal execution and management of prescribed fires are implemented if threatened fire-dependent species are to reach and maintain viable population levels.

Forest fires are also rare in other Nordic countries like Finland, where circa 1000 ha burn annually (Finnish statistical yearbook of forestry 2014). This is approximately 0.004% of their forest land, half of which can be attributed to prescribed fires. However, the major difference between Finland and Sweden is that the state performs the majority of prescribed fires (Peltola 2014), while in Sweden forestry companies are responsible for 86% of the area burnt as a result of prescribed fire. A large portion of these fires are on clearcuts and several studies have pointed out the limited benefits of burning clearcut forest sites (Hyvärinen et al. 2009; Johnson et al. 2014, Ranius et al. 2014; Heikkala et al. 2016). However, if retention trees are included in these burns, the value of these burns can increase substantially (Granath et al. unpublished).

The results also show that 40% of wildfires occur on low productive forest land, while no prescribed fires by forestry companies are executed on this forest type. In addition, historical occurrence of fire on low productive forest land, especially under dry conditions (chiefly mires), has been shown in several studies (Hörnberg et al. 1995; Hellberg et al. 2004). If the objective of prescribed fires is to emulate natural fire distribution in the forest landscape, it would be expected that they also include low productive forest areas. Although further studies exploring the importance of low productive forest land fires for preservation of biodiversity are needed, implementing prescribed fires on such forest land could be a cost-effective way to increase burnt area and thereby promote fire-dependent species.

### The use of databases for improving fire management

**Fig. 3:**
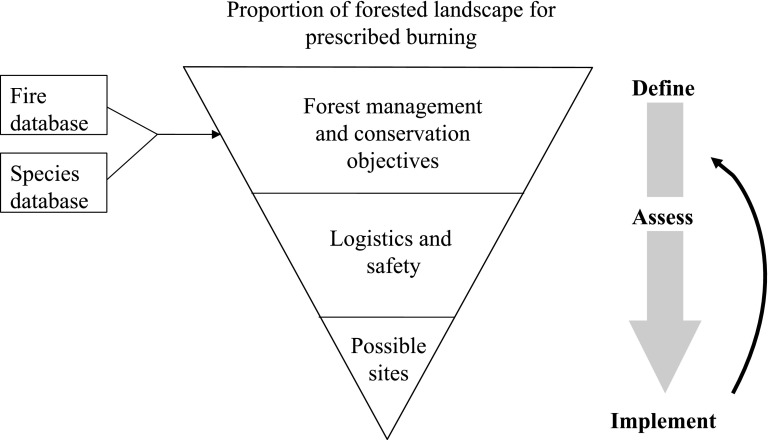
Conceptual diagram illustrating how databases can be incorporated in a management scheme to conduct prescribed fires based on a set of forest management and conservation objectives. The triangle illustrates how the forest landscape suitable for prescribed burning decreases as constraints are applied. With an informed assessment through databases, the final set of possible sites should be a cost-effective implementation that is more likely to reach the objectives. A feedback loop ensures an adaptive process responding to changes in incoming data and the need to change objectives due to limitations of suitable sites. See text also

Our study exemplifies how a national fire database can be used as a management tool for planning prescribed fires in the landscape. As landscape management is becoming increasingly complex, tools are needed to summarize complexity and help practitioners make informed decisions and implement optimal management to reach the set targets (Larson et al. 2013). In line with ideas of adaptive management (Walters and Hilborn 1978), we present a conceptual figure to aid the process of planning prescribed fires at a country level using a fire and a species occurrences database (Fig. 3). As prescribed burns vary in their effect on different species groups and ecosystem functions, it is important that the conservation objectives of the prescribed burning policy are defined first, and are in agreement with other management goals (Halme et al. 2013; Granath et al. unpublished). In the process of deciding the objectives for a particular area, landscape, or region, databases can be utilized to summarize information on species occurrences and fire history. Thereby, practitioners can define realistic objectives and make spatially explicit plans. Simple routines can be implemented for generating graphical outputs as presented here, and this is likely to be an effective way of obtaining relevant information especially for time-constrained practitioners. In this assessment process, the next step would involve identifying suitable sites that fulfill requirements on safety and logistics (e.g., road access). Here, knowledge on the species biology (e.g., life history traits) is vital for understanding habitat requirements and determining the temporal and spatial scales that are relevant (e.g., for dispersal) (Saint-Germain et al. 2008; Kouki et al. 2012). Finally, the actual implementation of prescribed burns has to be compared and evaluated with respect to the set objectives. Over time, as wildfires and species occurrences change, the stakeholders can learn to adapt their objectives and implementation methods, which is the basis of adaptive management [see Stankey et al. (2005) for a discussion about adaptive management]. This process relies on monitoring, e.g., of target species (either by stakeholders or by citizen), to provide up-to-date data that will inform future actions (Halme et al. 2013). Consequently, lack of data could be a hindrance to fully adapt this model, but even infrequent data could provide valuable prior information (Ruete 2015).

For our case studies of fire-dependent species, one of the goals was to preserve viable populations of the two species across their current distribution. By combining information from the databases (a fire database collated by us and the species occurrences database Swedish LifeWatch), we found that *G. lanuginosum*, an annual plant, is strongly tied to the occurrence of wildfires, with 80% of all records from locations where wildfires were recorded during the same time period. Of the prescribed fire between 2011 and 2015, the species was reported at only one site, indicating that few prescribed fires executed during the study period benefited *G. lanuginosum* and that today’s wildfires still are crucial for the species survival. The fact that *G. lanuginosum* is mainly tied to more nutrient-rich stands, which are less targeted for prescribed burning, may contribute to the negligible impact of prescribed burns on this species occurrence. The analysis of *S. substriatus* distribution showed that nearly half of the species finds were within 10.5 km of a prescribed fire, most of which were executed by forestry companies. In contrast to the plant *G. lanuginosum*, this species may have benefited from prescribed burns performed by the forestry companies in northern Sweden.

These case study species illustrate how potential fire sites can be identified using a database framework, combining species distributions as well as past mapped fires. Furthermore, it exemplifies how effects of prescribed fires can be evaluated in relation to occurrences of the species they are meant to benefit, as many species rely on regular fires within their potential distribution range (Granström 2001; Wikars 2004; Kouki et al. 2012). Moreover, since the population dynamics of many fire-dependent species is similar in structure to that of meta-populations (burnt areas being suitable patches only for a short period), there is theoretical basis for our applied framework. Future research efforts could be focused on developing meta-population models that explore under which fire dynamics (distance between fires, burnt area, frequency) different fire-dependent species (e.g., with contrasting dispersal capacity) remain viable/persist in the landscape. For practitioners, the approach presented in our study could potentially be implemented in the near future. A coherent management approach as shown in Fig. 3 would enable different stakeholders to plan fires in succession within the same landscape, making both planning and evaluation of prescribed fires more efficient. Since collaboration among stakeholders is necessary to reach the set conservation goals, but often hard to achieve given differing views and management cultures, a national database on fires (wildfires and prescribed) would simplify communication and coordinated efforts between stakeholders and possibly reduce complexity for practitioners.

## Conclusions

(i) The area attributed to wildfires in Sweden is small, yet seems to be important for the survival of some fire-dependent species; (ii) forestry certification is a driving force behind prescribed burns, and forest companies thereby play an important role in increasing the area of burned forest land; (iii) a large percentage of prescribed fires are clearcut burns and our knowledge is limited regarding their significance; (iv) fire and species occurrence databases can, together with adaptive management, aid in the planning of prescribed fires and increase the impact and efficiency of these fires; and (v) such an approach is technically easy to implement (as we showed), but potential conflicts in implementation among parties (e.g., reporting to a shared database) need to be resolved.
